# Plasma amino acids and oxylipins as potential multi-biomarkers for predicting diabetic macular edema

**DOI:** 10.1038/s41598-021-88104-y

**Published:** 2021-05-06

**Authors:** Sang Youl Rhee, Eun Sung Jung, Dong Ho Suh, Su Jin Jeong, Kiyoung Kim, Suk Chon, Seung-Young Yu, Jeong-Taek Woo, Choong Hwan Lee

**Affiliations:** 1 Department of Endocrinology and Metabolism, Kyung Hee University School of Medicine, 23 Kyungheedae-ro, Dongdaemun-gu, Seoul, 02447 Republic of Korea; 2 Department of Systems Biotechnology, Konkuk University, Seoul, Republic of Korea; 3 Department of Bioscience and Biotechnology, Konkuk University, 120 Neungdong-ro, Gwangjin-gu, Seoul, 05029 Republic of Korea; 4 Statistics Support Department, Kyung Hee University Medical Center Medical Science Research Institute, Seoul, Republic of Korea; 5 Department of Ophthalmology, Kyung Hee University School of Medicine, Seoul, Republic of Korea; 6 Research Institute for Bioactive-Metabolome Network, Konkuk University, Seoul, Republic of Korea

**Keywords:** Biomarkers, Endocrinology

## Abstract

To investigate the pathophysiologic characteristics of diabetic complications, we identified differences in plasma metabolites in subjects with type 2 diabetes (T2DM) with or without diabetic macular edema (DME) and a disease duration > 15 years. An cohort of older T2DM patients with prolonged disease duration was established, and clinical information and biospecimens were collected following the guidelines of the National Biobank of Korea. DME phenotypes were identified by ophthalmologic specialists. For metabolomics studies, propensity matched case and control samples were selected. To discover multi-biomarkers in plasma, non-targeted metabolite profiling and oxylipin profiling in the discovery cohort were validated in an extended cohort. From metabolomic studies, 5 amino acids (asparagine, aspartic acid, glutamic acid, cysteine, and lysine), 2 organic compounds (citric acid and uric acid) and 4 oxylipins (12-oxoETE, 15-oxoETE, 9-oxoODE, 20-carboxy leukotriene B4) were identified as candidate multi-biomarkers which can guide DME diagnosis among non-DME subjects. Receiver operating characteristic curves revealed high diagnostic value of the combined 5 amino acids and 2 organic compounds (AUC = 0.918), and of the 4 combined oxylipins (AUC = 0.957). Our study suggests that multi-biomarkers may be useful for predicting DME in older T2DM patients.

## Introduction

As the prevalence and disease duration of people with diabetes mellitus (DM) increase, the clinical significance of diabetic complications is also increasing^1,2^. In particular, diabetic retinopathy (DR) is specifically related to hyperglycemia, and is a very important complication that seriously impairs quality of life^3,4^. Active screening and treatment of DR can improve clinical outcomes in people with DM and improve their quality of life^5^. However, screening and appropriate treatment of DR has not been satisfactorily achieved compared to other complications^5–7^.

DR is generally classified as mild, moderate, severe non-proliferative DR (NPDR), and proliferative DR (PDR) depending on the degree of the risk of progression to neovascularization^8,9^. However, the most direct and important cause of visual impairment or blindness in people with DM is diabetic macular edema (DME)^10^. DME is a clinical manifestation characterized by retinal thickening and hard exudates that involve the macula^9^. These changes are known to be caused by damage to the blood-retinal barrier, which is composed of tight junctions involving vascular endothelial cells, and exudation of plasma proteins.

Differences exist between previous studies, but the prevalence of DME in people with DM is estimated to be approximately 10%^10^. In general, as the stage of DR advances, the prevalence of DME also tends to increase. However, DME can occur at any stage of DR, and the severity of DR does not exactly match the severity of DME^9,11,12^. Therefore, it is necessary to identify biomarkers that can screen for DME easily and accurately for effective clinical decision making. However, despite many efforts, studies in the field have not been very successful.

Recently, we performed metabolomic studies of diabetic complications using a biorepository organized for older adult subjects with type 2 DM (T2DM). In a previous study, we identified the clinical viability of novel biomarkers for effective prediction of DR^13^. Here, we conducted further such studies to identify novel DME-specific biomarkers that directly affect diabetic people with decreased vision and blindness.

## Research design and methods

### Subjects

This study was performed as part of the National Biobank of Korea project, using the baseline data of a prospective cohort collected from September 2014 to July 2015. The subjects of this cohort were older adult individuals with T2DM, with a disease duration of ≥ 15 years. There were no specific age restrictions in the initial design of the study, but participation over the age of 60 was encouraged.

Clinical information concerning the subjects was registered based on standardized methods for multi-center clinical data registration as approved by the Korean Diabetes Association, and biospecimens were gathered in accordance with the guidelines of the National Biobank of Korea^13,14^.

### Ethics statement

This study was approved by the institutional review board of Kyung Hee University Hospital (IRB No. KMC IRB 1428-04). All methods used in this study were carried out in accordance with the relevant guidelines and regulations of the institution in which the researchers are affiliated and comply with the Declaration of Helsinki. In addition, informed consent was obtained from all participants.

### Phenotyping of DME

The ophthalmologic status of each subject was evaluated through fundus photography (FF 540 Plus; Carl Zeiss Meditech, Jena, Germany) and by optical coherence tomography (HD-OCT; Carl Zeiss Meditech, Dublin, CA, USA). The method of evaluating the ophthalomologic status of study subjects has been described in detail in previous studies^4,15^.

Early Treatment Diabetic Retinopathy Study (ETDRS) criteria were used to diagnose DME, and one or more of the following were diagnosed as clinically significant macular edema; (1) Thickening of the retina ≤ 500 µm from the center of the macula, or (2) Hard exudates and adjacent retinal thickening ≤ 500 µm from the macular center, or (3) Zone of retinal thickening at least 1 disc area in size located ≤ 1 disc diameter from the center of the macula^8,9^. Two or more ophthalmologists diagnosed DME based on the results of examinations. If there was discordance between the ophthalmologists, they re-evaluated the results and agreed on the final phenotyping. In this study, even if one eye of subjects satisfies DME, we diagnosed it as a DME case. And when determining the phenotype of DME, there was no consideration of other ophthalmic diseases other than DR.

### Statistical analysis of clinical data

In this study, we evaluated and compared the clinical characteristics of subjects with and without DME, focusing on identifying the characteristics of subjects who did not have DME despite a long medical history of T2DM. Statistical analyses of the clinical characteristics were performed and compared between subjects with or without DME. After initial analysis, case and control sets were selected by propensity score matching (PSM) with similar clinical characteristics aside from DME, and corresponding samples were used for metabolomics studies. For PSM, variables such as age, sex, body mass index, blood pressure, glycated hemoglobin, blood chemistry, and comorbidities including DR were used. SAS software (v9.3; SAS Institute Inc., Cary, NC, USA) was used for all statistical analyses.

### Sample preparation for metabolomic studies

Metabolites were extracted from 200 μL of plasma. One milliliter of methanol containing an internal 2-chlorophenylalanine standard (1 mg/mL in water) was added to plasma samples and then homogenized using a mixer mill and sonicator for 10 min each, respectively. After homogenization, the suspension was held at 4 °C for 60 min, and then centrifuged at 20,000 × *g* and 4 °C for 10 min. The supernatant was filtered through a 0.2 μm polytetrafluoroethylene (PTFE) filter and dried using a speed vacuum concentrator (Modulspin 31; Biotron, Wonju, Korea). Dried samples were further oximated and silylated for gas chromatography time-of-flight mass spectrometry (GC–TOF–MS) analysis.

For extraction of oxylipins from plasma, Oasis-HLB cartridges were used. Prior to extraction, the cartridges were washed with ethyl acetate (2 mL), methanol (2 × 2 mL), and a solvent mixture (2 mL) of water and methanol (95:5 v/v) containing 0.1% acetic acid. After washing the cartridges, 200 μL of plasma was loaded onto the cartridges. After loading of samples, cartridges were washed with 1.5 mL of mixed solvent (water:methanol, 95:5 v/v, 0.1% acetic acid) under high vacuum. Then, cartridges were dried under low vacuum for 20 min. Oxylipins were eluted from the cartridges by adding 0.5 mL of methanol followed by 2 mL of ethyl acetate to tubes containing 6 μL of 30% glycerol in methanol as a trap solution. The solvents were dried using a speed vacuum concentrator. The extracts were reconstituted in methanol (10 mg/mL). After filtration, samples were analyzed using liquid chromatography (LC)-MS.

### Instrument analysis and data analysis for metabolomics studies

For metabolite profiling, GC-TOF–MS analysis was performed using an Agilent 7890 gas chromatography system (Agilent Technologies, Palo Alto, CA, USA) equipped with a Pegasus HT TOF MS (LECO Corp., St. Joseph, MI, USA) system. The detailed operational conditions for GC-TOF–MS analysis were identical to those described in our previous study^13^.

For oxylipin profiling, LC-triple-quadrupole-MS analysis was performed on a Nexera2 LC system (Shimadzu Corp., Kyoto, Japan) combined with a triple-quadrupole MS equipped with an electrospray source (LC–MS 8040, Shimadzu). Five microliters were injected into a Kinetex C18 column (100 × 2.1 mm, 2.6 µm, Phenomenex, Torrance, CA, USA) with a mobile phase containing 0.1% formic acid (solvent A) and acetonitrile containing 0.1% formic acid (solvent B) at a flow rate of 300 µL/min. The gradient was 5% solvent B for 1 min, and linearly increased from 5 to 100% over 9 min, and then decreased to 5% for 1 min. The MS platform was operated under the following conditions: capillary voltage -3000 V, capillary temperature 350ºC, vaporizer temperature 300ºC, sheath gas 3 L/min, ion sweep gas 2.0 Arb, Aux gas 10 Arb, and drying gas 8 L/min. The subsequent multiple reaction monitoring transitions used are summarized in Table S1.

Raw data from GC–TOF–MS analysis were preprocessed using several software packages. Detail concerning the processes of file conversion, alignment, and multivariate statistical analysis were identical to those described in our previous study^13^. Multivariate statistical analysis was performed by using SIMCA-P + (version 12.0; Umetrics, Umea, Sweden). The data were auto-scaled (unit variance scaling) and mean-centered in a column-wise fashion. Principal component analysis (PCA) and partial least squares–discriminant analysis (PLS–DA) were performed to compare each data set. The variables were selected based on variable importance to projection (VIP) values of the PLS–DA. R^2^X_(cum)_ and R^2^Y_(cum)_ are the cumulative modeled variation in X and Y matrix, respectively. Q^2^_(cum)_ is the cumulative predicted variation in Y matrix. *P* is *p* value obtained from cross-validation ANOVA of PLS-DA. The R^2^Y_(cum)_ value describes how well the data in the training set are mathematically reproduced (0 < x < 1, 1 = model with a perfect fit) and Q^2^_(cum)_ value indicates that x > 0.5, has a good predictive ability and x > 0.9, has excellent predictive ability. Significant differences were determined by student’s t-test using PASW Statistics 18 software (SPSS Inc., Chicago, IL, USA). Heat maps were rendered using the relative peak area of unique masses of metabolites by MeV software (http://www.tm4.org). Receiver operating characteristic (ROC) curves and logistic regression statistics were generated using Medcalc software (version 14.8.1; Medcalc Software, Mariakerke, Belgium).

## Results

### Clinical characteristics of the subjects after PSM

Of the 220 subjects who participated in the study, clinical data and specimens were collected from 198. After the withdrawal of 15 subjects, a total of 183 completed full ophthalmologic examinations. The mean age of the subjects was 66.8 years, mean T2DM duration was 22.6 years, and 50.3% were female. Of a total of 183 subjects who underwent ophthalmologic examinations, 124 (67.8%) were diagnosed with DR; and 46 (25.1%) were diagnosed with DME. PSM was performed, and 30 pairs of cases and controls with no significant differences in terms of clinical characteristics, except for the presence or absence of DME, were selected (Table S2). In addition, validation of the results derived from the discovery set was performed using a validation set of 43 pairs.

### Discovering multi-biomarkers of DME in plasma

**Figure 1:**
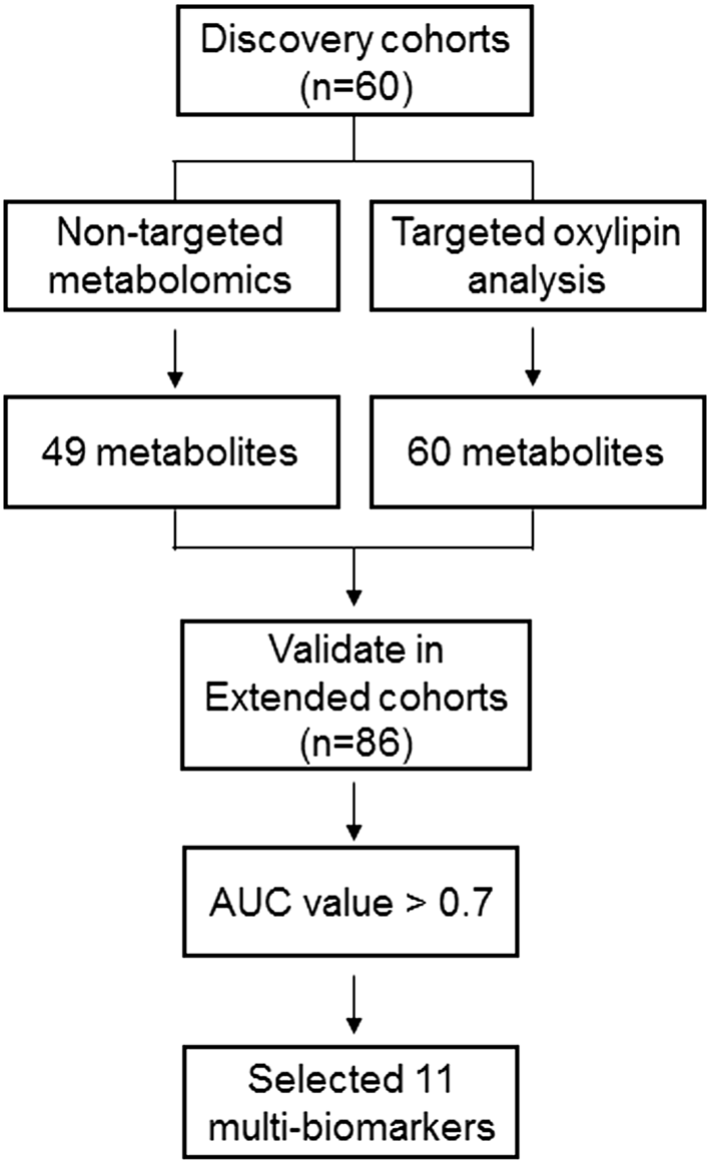
Schematic representation of experimental procedures used for investigating multi-biomarkers of diabetic macular edema (DME) in study subjects.

Based on metabolomics studies, we sought to discover multi-biomarkers in plasma which can help diagnose DME among DM subjects. A schematic diagram of the experimental processes involved is summarized in Fig. 1. First, we performed non-targeted metabolite and oxylipin profiling in the discovery cohort. Metabolites distinguishing subjects with and without DME were identified and selected as candidate metabolite biomarkers. The candidate metabolite biomarkers were confirmed in the extended cohort by comparing relative levels. Multi-biomarkers for discriminating DME and non-DME subjects were finally selected with the following qualifications: 1. Statistically significant discriminating metabolites from both the discovery and extended cohorts. 2. Metabolites exhibiting good discriminatory power for DME versus non-DME subjects, with an area under the curve (AUC) > 0.7 from discovery cohort.

### GC-TOF–MS analysis-based metabolite profiling and oxylipin analysis in plasma

**Figure 2:**
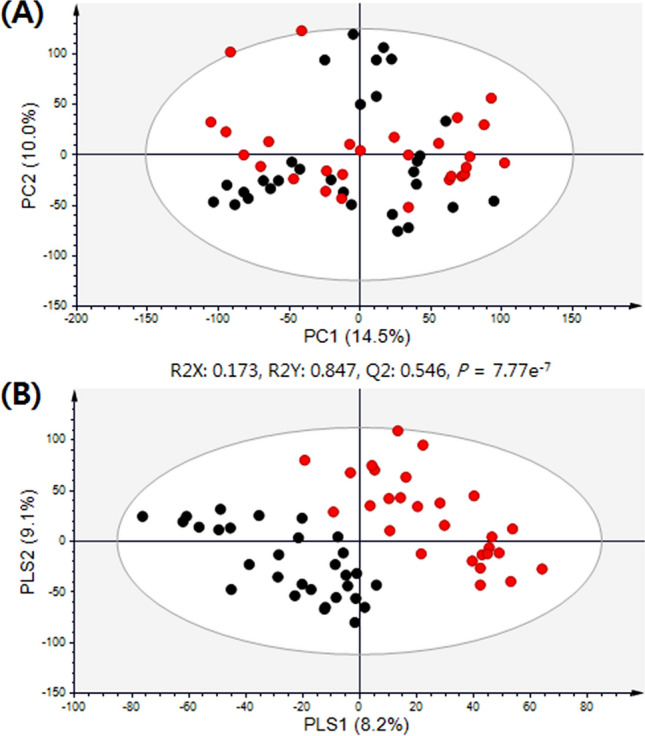
Principal component analysis (PCA) (**A**) and partial least squares discriminant analysis (PLS-DA) (**B**) score plots for candidate plasma markers in diabetic macular edema (DME) and non-DME subjects analyzed by GC–TOF–MS in discovery cohort. Black filled circle—non-DME group, red filled circle—DME group.

GC–TOF–MS analysis-based metabolite profiling was performed using plasma from discovery cohort subjects and multivariate statistical analysis (Fig. 2). In principal component analysis (PCA) score plots derived from metabolite profiling data sets using GC–TOF–MS analysis, the groups of DME and non-DME subjects were not clearly separated from each other. However, in the partial least squares–discriminant analysis (PLS-DA) model with supervised methods, these two groups were clearly distinguished from each other along with PLS1 (8.2%). The quality of the PLS-DA model was evaluated by R^2^Y_(cum)_ = 0.847, Q^2^_(cum)_ = 0.546, and by cross-validation analysis (7.77e^-7^), which signify a valid model. To select the metabolites responsible for the group separation, variable importance in projection (VIP) values > 0.7 of PLS-DA were applied. A total of 49 metabolites, including 19 amino acids, 14 organic compounds, 8 fatty acids and lipids, and 8 carbohydrates were identified as metabolites that differed between the DME and non-DME groups of subjects. In addition, a total of 60 oxylipins were identified by targeted analysis. Those included 36 arachidonic acid-, 9 docosahexaenoic acid (DHA)-, 6 eicosapentaenoic acid (EPA)-, and 9 linoleic acid-derived oxylipins. The relative metabolite levels were normalized to average values and visualized with heat maps (Figure S1A).

### Validation of plasma metabolite biomarkers for discriminating DME from non-DME cases

**Figure 3:**
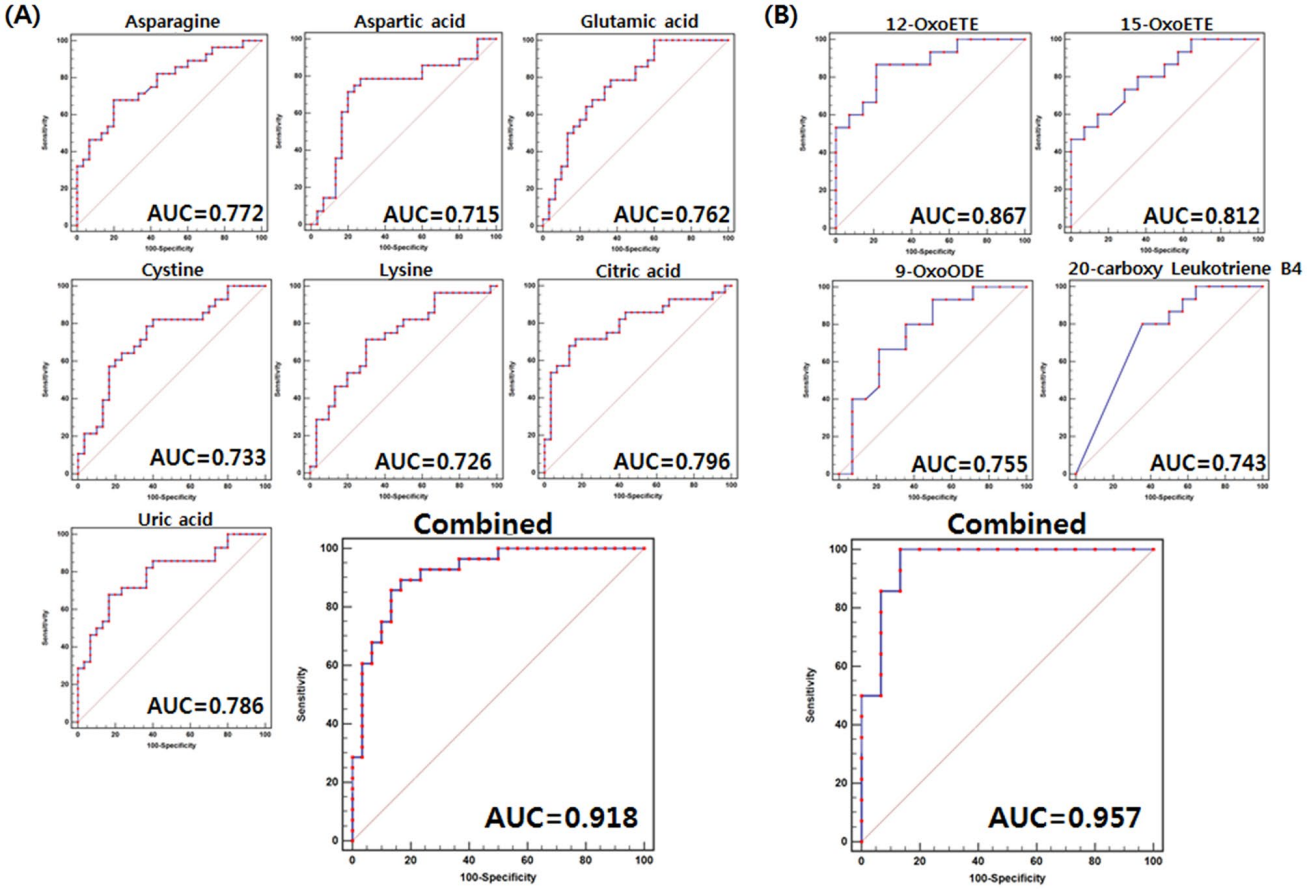
Receiver operating characteristic (ROC) curve of potential metabolite biomarkers distinguishing diabetic macular edema (DME) versus non-DME subjects, and combined ROC curves of those multi-biomarkers in discovery cohort. (**A**) 7 metabolites selected after GC–TOF–MS analysis-based metabolite profiling. (**B**) 4 oxylipins selected after lipid profiling analyzed by HPLC-triple-Q-MS. The ROC curves of each metabolite, and combined ROC curves, were overlain on single plots. The AUC values of each metabolite are shown inside the ROC curve.

To validate plasma metabolite biomarkers derived from the discovery cohort, we further performed multivariate analysis and oxylipin profiling using the extended cohort. The PCA and orthogonal PLS-DA (OPLS-DA) score plots revealed similar tendencies to those of the discovery cohort (Figure S2). However, the OPLS-DA model values were R^2^Y_(cum)_ = 0.693 and Q^2^_(cum)_ = 0.211, which indicated that the fitness and prediction accuracy of the model was lower than observed for the discovery cohort. The quality of the model was evaluated by cross-validation analysis (*p*-value = 0.0009). Metabolites distinguishing DME from non-DME subjects were selected according to the VIP value (> 0.7) of the extended cohort, and relative levels were visualized using heat maps (Figure S1B). By comparison of heat maps derived from the discovery and extended cohorts, relative metabolite levels between the groups of patients with DME and non-DME revealed similar tendencies. Multi-biomarkers for diagnosing DME patients were finally selected the following qualifications: 1. Statistically significant discriminant metabolites from both the discovery and extended cohorts. 2. Metabolites showing good discriminatory power for DME versus non-DME subjects, with an AUC > 0.7 from discovery cohort. Among the assigned metabolites, glutamic acid, cysteine, asparagine, aspartic acid, lysine, uric acid, malic acid, citric acid, nonanoic acid, 15-oxo-eicosatetraenoic acid (oxoETE), 12-oxoETE, 20-carboxy leukotriene B4, and 9-oxo-octadecaidenoic acid (oxoODE) exhibited statistically significant different levels between the groups of subjects with DME and non-DME in both the discovery and extended cohorts (Table S1, S3-S5). ROC curves were also generated for the 109 assigned metabolites using the relative metabolite levels of the experimental group in the discovery cohort (Table S1 and S3). Among them, metabolites which exhibited good discriminatory power for diabetic versus DME cases with an AUC > 0.7 included glutamic acid (0.762), cysteine (0.733), asparagine (0.772), aspartic acid (0.715), lysine (0.726), uric acid (0.786), citric acid (0.796), phenylacetic acid (0.810), 15-keto prostaglandin F2α (0.750), 15-keto prostaglandin E2 (0.719), 15-oxoETE (0.812), 12-oxoETE (0.867), 20-carboxy leukotriene B4 (0.743), 9-oxoODE (0.755), and ( ±) 9-hydroxyoctadecadienoic acid (HODE) or ( ±) 13-HODE (0.743). Finally, multi-biomarkers selected for distinguishing DME patients from non-DME subjects for diagnosis were asparagine (0.729-fold change), aspartic acid (0.782-fold), glutamic acid (0.653-fold), cysteine (0.666-fold), lysine (0.849-fold), citric acid (0.741-fold), uric acid (0.707-fold), 12-oxoETE (1.526-fold), 15-oxoETE (1.319-fold), 9-oxoODE (0.692-fold), and 20-carboxy leukotriene B4 (5.575-fold). A combination of these metabolites from GC-TOF–MS-based metabolite profiling, including asparagine, aspartic acid, glutamic acid, cysteine, lysine, citric acid, and uric acid, greatly improved the specificity of distinguishing DME subjects from non-DME cases, with a combined AUC value of 0.918 (Fig. 3). In addition, a combination of oxylipins, including 12-oxoETE, 15-oxoETE, 9-oxoODE, and 20-carboxy leukotriene B4, yielded a combined AUC value of 0.957, also demonstrating improved power in discriminating DME and non-DME subjects (Fig. 3). Multi-biomarkers that showed high prediction power in the discovery cohort showed the following AUC values in the extended cohort: Asparagine (0.666), aspartic acid (0.676), glutamic acid (0.733), cysteine (0.566), lysine (0.686), citric acid (0.684), uric acid (0.738), 12-oxoETE (0.784), 15-oxoETE (0.715), 9-oxoODE (0.711), and 20-carboxy leukotriene B4 (0.755) (Table S4 and S5). In addition, the combined ROC model of those of 5 amino acids and 2 organic acids, and 4 oxylipins were 0.852 and 0.877, respectively, which validated good predictive power in discriminating DME and non-DME subjects (Figure S3).

### Differences in metabolism according to generation of DME

**Figure 4:**
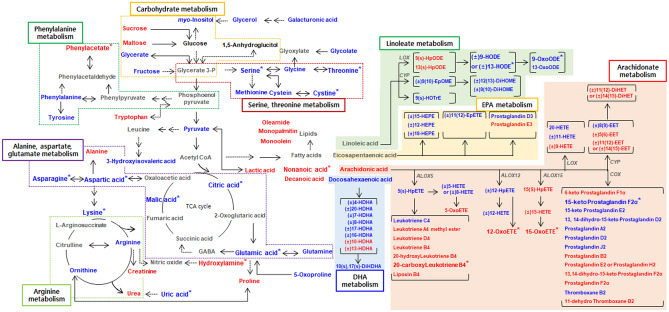
Schematic diagram of a proposed metabolic pathway using plasma metabolites derived from metabolite and lipid profiling of experimental groups including diabetic macular edema (DME) and non-DME subjects. Metabolites labelled with *blue characters* indicate that relative metabolite levels were lower in DME cases than in non-DME subjects. Metabolites labelled with *red character*s indicate that relative metabolite levels were higher in DME cases than in non-DME patients. Asterisks indicate statistically significant differences in levels of metabolites distinguishing DME and non-DME individuals (*p* < 0.05). The metabolic pathway was modified from the reported Kyoto Encyclopedia of Genes and Genomes pathway (KEGG, http://www.genome.jp/kegg/).

From plasma metabolome analysis of subjects with and without DME, various metabolites were selected as discriminatory factors, and we constructed a metabolic pathway to illustrate the relationships between metabolism and generation of DME (Fig. 4). In the pathway, carbohydrates, phenylalanine, alanine, aspartate, glutamate, arginine, and oxylipin metabolism (linoleate, eicosapentanoate, arachidonate and, docosahexaenoate metabolism) exhibited differences distinguishing patients with and without DME. In particular, metabolites such as serine, threonine, alanine, aspartate, and glutamate, and the tricarboxylic acid (TCA) metabolic cycle, were significantly decreased in subjects with DME compared with that in non-DME subjects. In the case of oxylipin metabolism, relative metabolite levels of oxylipin precursor fatty acids, such as linoleic, eicosapentanoic, arachidonic, and docosahexaenoic acids, were not significantly different between DME and non-DME subjects. However, the relative amounts of oxylipins produced from different precursor fatty acids did exhibit significant differences. Among them, most oxylipins involved in linoleate, EPA, and DHA metabolism showed relatively low metabolite levels in subjects with DME compared with that in non-DME subjects. In particular, in linoleate metabolism, oxylipins generated by lipoxygenase*,* peroxidase*,* and dehydrogenases such as (±) 9-HODE or (±) 13-HODE and 9-oxoODE were present at significantly lower levels in subjects with DME than in those without DME. In the case of arachidonate metabolism, a variety of oxylipins displayed increased and decreased metabolism due to DME compared those in non-DME subjects. Among these, levels of 20-carboxyleukotriene B4, 12-oxoETE, and 15-oxoETE, which are catalyzed by various enzymes, including hydroxylase, carboxylase, lipoxygenase, peroxidase, and dehydrogenase, were significantly elevated in DME subjects compared with those in non-DME subjects. On the other hand, 15-keto prostaglandin F2α, which is also generated by dehydrogenase activity, exhibited significantly decreased levels in subjects with DME.

## Discussion

Metabolomics represent a valuable approach for studying the pathophysiology of diverse disease states, including obesity^16^, DM^17^, and diabetic retinopathy^13^ by revealing differences in metabolic pathways according to disease status, and enabling the discovery of novel biomarkers for anticipating dysfunction and disease.

As reported in a previous study^13^, large numbers of metabolites are altered according to disease state, and these have been suggested as potential biomarker metabolites that may guide diagnosis by clinical examination of diabetes-related complications^18^. In particular, certain amino acids are known to have close relationships with insulin secretion by pancreatic β-cells in vivo. In this series of processes, various key enzymes and transporters are involved in the control of insulin secretion, such as glutamate dehydrogenase, aspartate and alanine aminotransferases, and the malate-aspartate shuttle^19^. According to metabolic pathway analysis of our results, amino acid metabolism related to the enzymes and transporters mentioned above were significantly altered with development of DME (Fig. 4). Those involved in amino acid metabolism, including alanine, aspartate, glutamate serine, and threonine were significantly decreased in subjects with DME compared with that in non-DME subjects (Figure S1A). Among these, cysteine and asparagine, aspartic, glutamic, and citric acids showed high (> 0.7) AUC values and were selected as plasma multi-biomarkers (Table S3). Various studies have reported relationships involving amino acids and diabetes. Zhou et al. reported that diabetes-related plasma amino acid alterations involving various amino acids, including serine, glutamine, aspartic acid, histidine, GABA, proline, lysine, leucine, and tryptophan, were significantly altered in subjects with DM compared with that in non-DM subjects^20^. In addition, another research team has also revealed changes to amino acid levels in diabetic patients and in non-diabetic cases. In particular, in diabetics, the relative levels of arginine, asparagine, glycine, serine, and threonine were decreased, with increased levels of alanine, isoleucine, leucine, valine compared to non-diabetics^21^. As is well known, various metabolites, including glucose, alanine, glutamine, leucine and arginine play important roles in insulin secretion. Glucose, alanine, and glutamine metabolism enhances the activity of the TCA cycle and generates metabolic secretion coupling factors such as ATP, Ca^2+^, and glutamate^22^. Overall, our results suggested that amino acid-related metabolism, among other factors affected by diabetes, may influence the development of DME.

Recently, a number of studies have suggested a close association between diabetes and diabetes-related complications, and polyunsaturated fatty acids (PUFAs) metabolism^23–25^. For these reasons, the importance of lipid profiling has emerged. However, only a small number of studies have investigated metabolic biomarkers for anticipating DME in DM subjects. In particular, correlations involving DME and oxylipins in DM subjects remain unknown.

Oxylipins are oxygenated lipids which are synthesized via metabolism of PUFAs, including linoleic, alpha-linolenic, arachidonic, eicosapentaenoic, and docosahexaenoic acids by various enzymatic reactions such as lipoxygenase (LOX), cyclooxygenase (COX), and cytochrome p450s (CYPs). In particular, long chain PUFAs are abundant in retina^25^. In addition, *ω-*3 PUFAs and *ω-*6 PUFAs can exert anti- and pro-inflammatory effects in humans, respectively^26^. Based on our results, 4 oxylipins, including 12-oxoETE, 15-oxoETE, 9-oxoODE, and 20-carboxy leukotriene B4 derived from *ω-*6 PUFAs (arachidonic acid and linoleic acid), were selected as plasma multi-biomarkers (Fig. 3). Among these, 12-oxoETE, 15-oxoETE, and 20-carboxy leukotriene B4 derived from arachidonic acid via the LOX pathway, and their relative metabolic levels were significantly increased in subjects with DME compared with that in non-DME subjects (Fig. 4). In the case of 9-oxoODE, which is derived from the metabolism of linoleic acid via the LOX pathway, 9-oxoODE levels were significantly diminished in subjects with DME compared with that in non-DME subjects. Intriguingly, previous researchers have described the expression of LOX and its relationship with diabetes in that 12-LOX and 15-LOX play critical roles in the modulation of inflammation at multiple checkpoints during diabetes development^23^. In addition, the 5-LOX leukotriene biosynthesis pathway is enhanced in inflammatory states in white adipose tissue in T2DM women^24^. Hence, we propose that increased levels of oxylipins, which are metabolites produced by LOX catalytic activity, may be the result or cause of development of DME.

The key findings of the present study were the identification and suggestion of plasma multi-biomarker metabolites distinguishing subjects with DME from those with DM. In a previous study, we identified novel biomarkers for the effective prediction of diabetic retinopathy, and their value as biomarkers was effectively supported by the AUC value of the ROC curves^13^. In the current study of discovery cohort, the combined AUCs of the 5 amino acids and 2 organic acids, and 4 oxylipins were 0.918 and 0.957, respectively (Fig. 3). Considering the predictive power of the ROC model, a combination of these multiple biomarkers could enable the distinction of DME patients from diabetic controls. Good predictive power of the ROC model also validated in extended cohort that the combined AUCs of the 5 amino acids and 2 organic acids, and 4 oxylipins were 0.852 and 0.877, respectively (Figure S3). Indeed, many research teams have conducted clinical trials involving the disease using a combination of metabolites, and have confirmed positive efficacy. Several studies have been reported regarding the ingestion of protein hydrolysates and amino acid mixtures with carbohydrates improves insulin secretion and plasma glucose disposal in T2DM patients^27,28^. In addition, long term (60 weeks) dietary supplementation with amino acid mixtures, including leucine, lysine, isoleucine, valine, threonine, cysteine, histidine, phenylalanine, methionine, tyrosine, and tryptophan, significantly improve insulin sensitivity in poorly controlled older adult subjects with T2DM^29^. Most recent, the importance of stabilization of epoxygenated fatty acids is emerged in therapeutic relevance in eye disease. Those epoxygenated fatty acids were derived from *ω-*3 PUFAs via CYP pathway and known to have anti-inflammatory and anti-angiogenic effects^30^. Capozzi et al. revealed that of elevation of epoxygenated fatty acid levels, such as 11,12-epoxyeicosatienoic acid (EET) and 19,20-epoxydocosapentaenoic acid (EDP) by exogenous addition, inhibits VCAM-1 and ICAM-1 expression and protein levels, and induces leukostasis in a mouse model of acute retinal inflammation, which may be related to TNF-α-induced inflammation in retinal vascular diseases^31^. In addition, Chistyakov et al. established the baseline patterns of oxylipins in aqueous humor and tear fluid for used as reference in ocular inflammation studies^32^. Through these, it could be suggested that it is important to control the metabolism of oxylipin, and delivering important metabolites or regulating compounds through eye drops may be effective. Since the multi-biomarkers identified in this study may also have the effect of improving DME, additional studies to confirm their efficacy in animal and clinical studies are needed.

In conclusion, in this study, we undertook a comprehensive metabolomics approach to discover candidate metabolic multi-biomarkers for diagnosis of DME from non-DME patients and performed metabolic pathway analysis to examine mechanisms related to the development of DME. We revealed that certain plasma amino acids (asparagine, aspartic acid, glutamic acid, cysteine, and lysine), organic compounds (citric acid and uric acid), and oxylipins (12-oxoETE, 15-oxo-ETE, 9-oxoODE, and 20-carboxy leukotriene B4) could be used as indicators for establishing a means of long-term prognosis associated with DME in long-standing T2DM patients.

## Supplementary Information

Supplementary Information.
