# Supplementary material for: Plasma amino acids and oxylipins as potential multi-biomarkers for predicting diabetic macular edema
Source: Sci Rep. 2021 May 6;11:9727. doi: 10.1038/s41598-021-88104-y (PMC8102569; doi:10.1038/s41598-021-88104-y)

**Plasma amino acids and oxylipins as potential multi-biomarkers for predicting diabetic macular edema**

Sang Youl Rhee^1*^, Eun Sung Jung^2*^, Dong Ho Suh^3^, Su Jin Jeong^4^, Kiyoung Kim^5^, Suk Chon^1^, Seung-Young Yu^5^, Jeong-Taek Woo^1**^, Choong Hwan Lee^2,3,6**^

^1^Department of Endocrinology and Metabolism, Kyung Hee University School of Medicine, Seoul, Korea

^2^Department of Systems Biotechnology, Konkuk University, Seoul, Korea

^3^Department of Bioscience and Biotechnology, Konkuk University, Seoul, Korea

^4^Statistics Support Department, Kyung Hee University Medical Center Medical Science Research Institute, Seoul, Korea

^5^Department of Ophthalmology, Kyung Hee University School of Medicine, Seoul, Korea

^6^Research Institute for Bioactive-Metabolome Network, Konkuk University, Seoul, Korea

**Supplementary Tables**

**Table S1.** Plasma oxylipins identified in DME and non-DME subjects based on lipid profiling analyzed by HPLC-triple-Q-MS in discovery cohort.

| **No.** | **RT**  **(min)** | **Molecular**  **weight** | **Product ion (*m/z*)** | **Metabolites** | **AUC** | **Fold Change (DME/non-DME)** | **t-test (*p*-value)** |
| --- | --- | --- | --- | --- | --- | --- | --- |
| 50 | 7.11 | 320.5 | 275.3 | 20-HETE | 0.598 | 0.301 | 0.2217 |
| 51 | 3.96 | 352.5 | 315.25 | 15-keto Prostaglandin F2α | 0.750 | 0.341 | 0.0134 |
| 52 | 3.91 | 350.5 | 331.2 | 15-keto Prostaglandin E2 | 0.719 | 0.491 | 0.1439 |
| 53 | 3.35 | 370.5 | 195.2 | Thromboxane B2 | 0.598 | 0.643 | 0.4696 |
| 54 | 4.32 | 352.5 | 207.2 | 13,14-dihydro-15-keto Prostaglandin D2 | 0.617 | 0.746 | 0.5744 |
| 55 | 5.11 | 336.5 | 59.1 | 5(S)-HpETE | 0.624 | 0.752 | 0.2161 |
| 56 | 7.00 | 320.5 | 167.2 | (±) 11-HETE | 0.624 | 0.775 | 0.3183 |
| 57 | 7.24 | 320.5 | 155.15 | (±) 8 (9)-EET | 0.588 | 0.783 | 0.4773 |
| 58 | 7.07 | 320.5 | 179.2 | (±) 12-HETE | 0.579 | 0.804 | 0.3520 |
| 59 | 7.29 | 320.5 | 301.25 | (±) 5-HETE or (±) 8-HETE | 0.586 | 0.805 | 0.4086 |
| 60 | 4.94 | 336.5 | 317.3 | (±) 12-HpETE | 0.505 | 0.848 | 0.5845 |
| 61 | 3.74 | 625.8 | 189.15 | Leukotriene C4 | 0.505 | 0.853 | 0.6197 |
| 62 | 4.48 | 334.5 | 315.25 | Prostaglandin A2 | 0.526 | 0.878 | 0.8179 |
| 63 | 3.88 | 352.5 | 189.25 | Prostaglandin D2 | 0.586 | 0.915 | 0.7283 |
| 64 | 4.61 | 334.5 | 271.3 | Prostaglandin J2 | 0.524 | 0.991 | 0.9806 |
| 65 | 8.02 | 318.5 | 203.25 | 5-OxoETE | 0.548 | 1.041 | 0.9355 |
| 66 | 4.94 | 338.5 | 319.3 | (±) 11(12)-DiHET or (±) 14 (15)-DiHET | 0.557 | 1.060 | 0.8009 |
| 67 | 7.14 | 320.5 | 163.3 | (±) 5(6)-EET | 0.574 | 1.115 | 0.7319 |
| 68 | 3.64 | 332.5 | 315.15 | Leukotriene A4 methyl ester | 0.519 | 1.140 | 0.3959 |
| 69 | 4.08 | 496.7 | 177.1 | Leukotriene D4 | 0.605 | 1.203 | 0.5726 |
| 70 | 5.17 | 336.5 | 195.2 | Leukotriene B4 | 0.562 | 1.220 | 0.5217 |
| 71 | 6.81 | 320.5 | 219.2 | (±) 15-HETE | 0.655 | 1.274 | 0.3533 |
| 72 | 7.24 | 318.5 | 113.1 | 15-OxoETE | 0.812 | 1.319 | 0.0015 |
| 73 | 7.28 | 320.5 | 151.2 | (±) 9-HETE | 0.602 | 1.373 | 0.3123 |
| 74 | 4.50 | 334.5 | 235.15 | Prostaglandin B2 | 0.595 | 1.453 | 0.4526 |
| 75 | 3.99 | 352.5 | 271.3 | Prostaglandin E2 or Prostaglandin H2 | 0.648 | 1.463 | 0.3641 |
| 76 | 7.28 | 320.5 | 257.25 | (±) 11(12)-EET or (±) 14(15)-EET | 0.686 | 1.465 | 0.0815 |
| 77 | 4.01 | 352.5 | 333.25 | 20-hydroxy Leukotriene B4 | 0.602 | 1.479 | 0.4298 |
| 78 | 6.07 | 318.5 | 299.85 | 12-OxoETE | 0.867 | 1.526 | 0.0012 |
| 79 | 3.38 | 368.5 | 349.25 | 11-dehydro Thromboxane B2 | 0.538 | 1.578 | 0.4480 |
| 80 | 4.50 | 354.5 | 113.1 | 13,14-dihydro-15-keto Prostaglandin F2α | 0.571 | 2.816 | 0.3957 |
| 81 | 3.98 | 352.5 | 221.25 | Lipoxin B4 | 0.586 | 4.364 | 0.1504 |
| 82 | 3.02 | 366.5 | 347.25 | 20-carboxy Leukotriene B4 | 0.743 | 5.575 | 0.0121 |
| 83 | 3.72 | 354.5 | 309.25 | Prostaglandin F2α | 0.543 | 6.221 | 0.2511 |
| 84 | 3.25 | 370.5 | 163.2 | 6-keto Prostaglandin F1α | 0.536 | 13657142858.071 | 0.3092 |
| 85 | 4.88 | 336.5 | 273.25 | 15 (S)-HpETE | 0.607 | 22557142857.929 | 0.0661 |
| 86 | 7.65 | 344.5 | 101.05 | (±) 4-HDHA | 0.690 | 0.757 | 0.0752 |
| 87 | 7.16 | 344.5 | 325.25 | (±)2 0-HDHA | 0.669 | 0.769 | 0.1138 |
| 88 | 7.39 | 344.5 | 141.15 | (±) 7-HDHA | 0.524 | 0.895 | 0.8666 |
| 89 | 7.38 | 344.5 | 189.25 | (±) 8-HDHA | 0.650 | 0.908 | 0.6225 |
| 90 | 7.06 | 344.5 | 245.25 | (±) 17-HDHA | 0.531 | 0.923 | 0.9411 |
| 91 | 7.06 | 344.5 | 233.25 | (±) 16-HDHA | 0.595 | 0.939 | 0.7086 |
| 92 | 5.01 | 360.5 | 153.2 | 10(S),17(S)-DiHDHA | 0.558 | 0.966 | 0.6005 |
| 93 | 7.24 | 344.5 | 153.15 | (±) 10-HDHA | 0.505 | 1.130 | 0.5592 |
| 94 | 7.22 | 344.5 | 281.25 | (±) 13-HDHA | 0.538 | 1.187 | 0.3507 |
| 95 | 6.40 | 318.5 | 219.2 | (±) 15-HEPE | 0.617 | 0.812 | 0.4395 |
| 96 | 6.60 | 318.5 | 255.3 | (±) 11(12)-EpETE | 0.633 | 0.887 | 0.5893 |
| 97 | 6.54 | 318.5 | 179.2 | (±) 12-HEPE | 0.550 | 0.891 | 0.6076 |
| 98 | 3.88 | 350.5 | 233.2 | Prostaglandin D3 | 0.634 | 0.898 | 0.9934 |
| 99 | 6.52 | 318.5 | 299.25 | (±) 18-HEPE | 0.567 | 0.924 | 0.7107 |
| 100 | 3.77 | 350.5 | 269.25 | Prostaglandin E3 | 0.518 | 1.417 | 0.3548 |
| 101 | 7.53 | 294.4 | 185.2 | 9-OxoODE | 0.755 | 0.692 | 0.0225 |
| 102 | 7.26 | 294.4 | 113.15 | 13-OxoODE | 0.617 | 0.480 | 0.1317 |
| 103 | 5.18 | 314.5 | 183.25 | (±) 12(13)-DiHOME | 0.643 | 0.822 | 0.2681 |
| 104 | 6.88 | 296.5 | 277.4 | (±) 9(10)-EpOME | 0.624 | 0.838 | 0.3235 |
| 105 | 5.30 | 314.5 | 201.2 | (±) 9(10)-DiHOME | 0.586 | 0.914 | 0.7023 |
| 106 | 7.76 | 296.5 | 301.05 | (±) 9-HODE or (±) 13-HODE | 0.743 | 0.924 | 0.0195 |
| 107 | 6.15 | 294.4 | 275.25 | 9(S)-HOTrE | 0.610 | 0.931 | 0.7274 |
| 108 | 6.60 | 312.4 | 183.1 | 13(S)-HpODE | 0.548 | 1.037 | 0.8402 |
| 109 | 6.56 | 312.4 | 220.1 | 9(S)-HpODE | 0.567 | 1.067 | 0.8127 |

**Table S2.** Clinical characteristics of subjects after PSM.

| **Category** | **Variables** | **Discovery cohort (30 pairs)** | | | **Extended cohort (43 pairs)** | | |
| --- | --- | --- | --- | --- | --- | --- | --- |
|  |  | No ME | ME | *p* | No ME | ME | *p* |
| Clinical characteristics | Gender (female, pair) | 9 | 2 | 1.000 | 16 | 10 | 1.000 |
|  | DM duration (yr) | 22.10 ± 6.78 | 23.70 ± 6.95 | 0.329 | 21.84 ± 7.27 | 23.81 ± 6.37 | 0.088 |
|  | Age (yr) | 66.07 ± 8.71 | 61.73 ± 10.51 | 0.068 | 65.02 ± 8.81 | 62.33 ± 10.18 | 0.143 |
|  | Height (cm) | 159 ± 9.21 | 158.73 ± 8.2 | 0.913 | 159.57 ± 9.21 | 158.63 ± 8.51 | 0.731 |
|  | Weight (kg) | 61.87 ± 8.8 | 61.91 ± 8.77 | 0.988 | 62.42 ± 9.06 | 61.17 ± 8.84 | 0.456 |
|  | BMI (kg/m^2^) | 24.48 ± 2.96 | 24.65 ± 3.7 | 0.834 | 24.49 ± 2.83 | 24.37 ± 3.61 | 0.920 |
|  | Waist circumference (cm) | 89.04 ± 7.79 | 89.58 ± 11.84 | 0.819 | 88.58 ± 7.41 | 88.27 ± 10.91 | 0.781 |
|  | Systolic blood pressure (mm Hg) | 123.17 ± 14.05 | 123.6 ± 14.13 | 0.897 | 125.44 ± 13.85 | 124.19 ± 14.41 | 0.515 |
|  | Diastolic blood pressure (mm Hg) | 69.37 ± 8.04 | 68.43 ± 10.23 | 0.635 | 70.49 ± 8.15 | 68.21 ± 9.89 | 0.235 |
|  | HbA1c (%) | 8.37 ± 1.92 | 8.35 ± 1.56 | 0.974 | 8.20 ± 1.76 | 8.42 ± 1.45 | 0.586 |
|  | Fasting plasma glucose (mg/dL) | 166.7 ± 71.48 | 159.93 ± 68.98 | 0.708 | 169.51 ± 81.48 | 166.67 ± 67.11 | 0.976 |
|  | Total cholesterol (mg/dL) | 178.1 ± 41.35 | 165.5 ± 33.46 | 0.265 | 177.58 ± 38.1 | 163.65 ± 31.72 | 0.169 |
|  | Triglyceride (mg/dL) | 147.33 ± 111.43 | 154.2 ± 85.01 | 0.782 | 139.49 ± 105.01 | 143.79 ± 79.15 | 0.465 |
|  | LDL cholesterol (mg/dL) | 107.8 ± 33.45 | 96.23 ± 28.89 | 0.237 | 105.3 ± 31.65 | 94.44 ± 27.8 | 0.220 |
|  | HDL cholesterol (mg/dL) | 52.13 ± 19.49 | 48.63 ± 10.9 | 0.377 | 53.6 ± 18.12 | 49.86 ± 14.87 | 0.480 |
|  | BUN (mg/dL) | 21.93 ± 10.5 | 19.33 ± 6.81 | 0.213 | 22.95 ± 15.43 | 21.44 ± 10.77 | 0.596 |
|  | Creatinine (mg/dL) | 0.89 ± 0.43 | 0.88 ± 0.37 | 0.969 | 1.12 ± 1.29 | 1.03 ± 0.72 | 0.735 |
|  | Creatinine Clearance (mL/min/1.73 m^2^) | 91.86 ± 38.5 | 90.16 ± 33.6 | 0.828 | 89.08 ± 37.99 | 84.85 ± 34.91 | 0.664 |
|  | AST (IU/L) | 23.7 ± 5.09 | 21.83 ± 6.81 | 0.194 | 22.84 ± 5.08 | 21.6 ± 6.19 | 0.206 |
|  | ALT (IU/L) | 16.43 ± 3.47 | 16.1 ± 5.31 | 0.796 | 17.14 ± 5.1 | 15.51 ± 5.41 | 0.157 |
|  | GGT (IU/L) | 23.03 ± 12 | 23 ± 8.55 | 0.987 | 29 ± 23.96 | 21.12 ± 8.37 | 0.094 |
|  | ALP (IU/L) | 80.87 ± 27.6 | 86.63 ± 22.98 | 0.362 | 83.95 ± 28.73 | 85.47 ± 22.27 | 0.532 |
| History of macrovascular complication | Hypertension (pair) | 5 | 20 | 1.000 | 7 | 29 | 1.000 |
|  | Dyslipidemia (pair) | 11 | 10 | 0.480 | 14 | 14 | 1.000 |
|  | Myocardial infarction (pair) | 0 | 0 | - | 0 | 0 | - |
|  | Angina (pair) | 3 | 0 | - | 4 | 0 | - |
|  | Heart failure (pair) | 0 | 0 | - | 1 | 0 | - |
|  | Atrial fibrillation (pair) | 0 | 0 | - | 2 | 0 | - |
|  | Any stroke (pair) | 2 | 1 | 0.683 | 3 | 1 | 1.000 |
| History of microvascular complication | Retinopathy (pair) | 2 | 14 | 0.010 | 3 | 21 | 0.002 |
|  | Glaucoma (pair) | 3 | 1 | 1.000 | 3 | 1 | 0.228 |
|  | Cataract (pair) | 6 | 11 | 1.000 | 9 | 15 | 1.000 |
|  | Chronic Kidney Disease (pair) | 5 | 1 | 1.000 | 7 | 3 | 1.000 |
|  | Peripheral neuropathy (pair) | 8 | 7 | 0.814 | 11 | 12 | 0.556 |
|  | Autonomic neuropathy (pair) | 6 | 5 | 1.000 | 6 | 6 | 0.789 |
| Current Medications | Metformin (pair) | 4 | 18 | 1.000 | 7 | 20 | 0.803 |
|  | Sulfonylurea (pair) | 7 | 11 | 0.773 | 13 | 12 | 0.383 |
|  | DPP-4 inhibitor (pair) | 8 | 2 | 0.579 | 10 | 3 | 0.628 |
|  | Meglitinide (pair) | 1 | 0 | 1.000 | 2 | 0 | 0.683 |
|  | Thiazolidinedione (pair) | 1 | 0 | 1.000 | 2 | 0 | 1.000 |
|  | SGLT-2 inhibitor (pair) | 0 | 0 | - | 0 | 0 | - |
|  | Alpha glucosidase inhibitor (pair) | 0 | 0 | - | 0 | 0 | - |
|  | Rapid acting insulin (pair) | 5 | 3 | 1.000 | 8 | 4 | 1.000 |
|  | Basal insulin (pair) | 9 | 5 | 1.000 | 13 | 8 | 0.677 |
|  | Pre-mixed insulin (pair) | 3 | 2 | 1.000 | 4 | 2 | 0.547 |
|  | GLP-1 agonist (pair) | 0 | 0 | - | 0 | 0 | - |
|  | Angiotensin Receptor Blocker (pair) | 8 | 9 | 0.789 | 11 | 12 | 0.480 |
|  | Angiotension Converting Enzyme inhibitor (pair) | 0 | 0 | 0.074 | 2 | 0 | 0.289 |
|  | Calcium channel blocker (pair) | 6 | 2 | 1.000 | 9 | 3 | 0.823 |
|  | Diuretics (pair) | 5 | 1 | 0.221 | 6 | 1 | 0.505 |
|  | Beta blocker (pair) | 4 | 1 | 0.683 | 6 | 3 | 0.289 |
|  | Statin (pair) | 10 | 8 | 1.000 | 12 | 12 | 1.000 |
|  | Fibrate (pair) | 1 | 0 | 1.000 | 1 | 0 | - |
|  | Aspirin (pair) | 5 | 1 | 1.000 | 8 | 2 | 0.387 |
|  | Clopidogrel (pair) | 4 | 0 | 0.683 | 6 | 0 | 0.752 |
|  | Cilostazol (pair) | 8 | 4 | 0.387 | 11 | 9 | 0.211 |

expressed as mean ± SD, or n (%).by Paired sample t-test, or McNemar’s test.

ME, macular edema; DM, diabetes mellitus; BMI, body mass index; LDL, Low density lipoprotein; HDL, high density lipoprotein; BUN, blood urea nitrogen; AST, aspartate aminotransferase; ALT, alanine aminotransferase; GGT, gamma-glutamyl transferase; ALP, alkaline phosphatase; CAG, coronary angiography; DPP, dipeptidylpeptidase; SGLT, sodium-glucose transporter.

**Table S3.** Plasma metabolites discriminating DME and non DME subjects based on metabolite profiling analyzed by GC-TOF-MS in discovery cohort.

| **No.** | **RT**  **(min)** | **Unique**  **Mass** | **Metabolites** | **AUC** | **Fold Change (DME/non-DME)** | **t-test (*p*-value)** |
| --- | --- | --- | --- | --- | --- | --- |
| 1 | 9.75 | 220 | Cysteine | 0.658 | 0.645 | 0.0934 |
| 2 | 10.23 | 246 | Glutamic acid | 0.762 | 0.653 | 0.0003 |
| 3 | 14.76 | 218 | Cystine | 0.733 | 0.666 | 0.0015 |
| 4 | 10.66 | 116 | Asparagine | 0.772 | 0.729 | 0.0006 |
| 5 | 9.45 | 232 | Aspartic acid | 0.715 | 0.782 | 0.0130 |
| 6 | 8.31 | 219 | Threonine | 0.670 | 0.819 | 0.0296 |
| 7 | 11.72 | 142 | Ornithine | 0.682 | 0.820 | 0.0504 |
| 8 | 9.51 | 156 | 5-Oxoproline | 0.676 | 0.826 | 0.1042 |
| 9 | 10.19 | 142 | Arginine | 0.620 | 0.842 | 0.2285 |
| 10 | 8.06 | 204 | Serine | 0.692 | 0.843 | 0.0463 |
| 11 | 12.45 | 174 | Lysine | 0.726 | 0.849 | 0.0041 |
| 12 | 11.12 | 156 | Glutamine | 0.621 | 0.857 | 0.0867 |
| 13 | 9.45 | 176 | Methionine | 0.602 | 0.878 | 0.0985 |
| 14 | 7.58 | 174 | Glycine | 0.664 | 0.880 | 0.0837 |
| 15 | 12.58 | 218 | Tyrosine | 0.536 | 0.941 | 0.4090 |
| 16 | 10.33 | 218 | Phenylalanine | 0.576 | 0.950 | 0.3471 |
| 17 | 5.56 | 116 | Alanine | 0.542 | 1.080 | 0.4789 |
| 18 | 7.50 | 142 | Proline | 0.524 | 1.098 | 0.3269 |
| 19 | 14.46 | 204 | Tryptophan | 0.562 | 1.245 | 0.2263 |
| 20 | 9.04 | 218 | Aminomalonic acid | 0.693 | 0.693 | 0.0051 |
| 21 | 13.64 | 441 | Uric acid | 0.786 | 0.707 | 0.0001 |
| 22 | 11.76 | 273 | Citric acid | 0.796 | 0.741 | 0.0001 |
| 23 | 9.18 | 233 | Malic acid | 0.688 | 0.749 | 0.0169 |
| 24 | 7.80 | 189 | Glyceric acid | 0.638 | 0.803 | 0.0735 |
| 25 | 12.64 | 333 | Galacturonic acid | 0.620 | 0.859 | 0.3721 |
| 26 | 5.05 | 174 | Pyruvic acid | 0.566 | 0.894 | 0.3430 |
| 27 | 5.29 | 177 | Glycolic acid | 0.590 | 0.914 | 0.2367 |
| 28 | 7.32 | 299 | Phosphoric acid | 0.519 | 1.049 | 0.4954 |
| 29 | 5.15 | 117 | Lactic acid | 0.562 | 1.059 | 0.2686 |
| 30 | 5.70 | 133 | Hydroxylamine | 0.640 | 1.155 | 0.0318 |
| 31 | 7.06 | 189 | Urea | 0.630 | 1.223 | 0.0749 |
| 32 | 9.79 | 115 | Creatinine | 0.652 | 1.239 | 0.0946 |
| 33 | 7.51 | 164 | Phenylacetic acid | 0.810 | 1.393 | 0.0000 |
| 34 | 16.12 | 91 | Docosahexaenoic acid | 0.600 | 0.805 | 0.0797 |
| 35 | 6.61 | 131 | 3-Hydroxyisovaleric acid | 0.528 | 0.929 | 0.3489 |
| 36 | 15.35 | 131 | Oleamide | 0.560 | 1.037 | 0.4464 |
| 37 | 17.07 | 397 | Monoolein | 0.518 | 1.039 | 0.7291 |
| 38 | 16.23 | 371 | Monopalmitin | 0.602 | 1.135 | 0.1011 |
| 39 | 8.01 | 215 | Nonanoic acid | 0.671 | 1.171 | 0.0332 |
| 40 | 8.86 | 229 | Decanoic acid | 0.691 | 1.192 | 0.0881 |
| 41 | 15.03 | 117 | Arachidonic acid | 0.530 | 1.424 | 0.3116 |
| 42 | 13.62 | 217 | myo-Inositol | 0.619 | 0.807 | 0.1552 |
| 43 | 7.25 | 117 | Glycerol | 0.592 | 0.911 | 0.2024 |
| 44 | 12.26 | 103 | Fructose | 0.588 | 0.918 | 0.3479 |
| 45 | 12.38 | 205 | Glucose | 0.520 | 0.999 | 0.9894 |
| 46 | 12.52 | 205 | Glucose | 0.520 | 1.022 | 0.6353 |
| 47 | 12.00 | 191 | 1,5-Anhydroglucitol | 0.531 | 1.028 | 0.9199 |
| 48 | 17.24 | 361 | Maltose | 0.612 | 1.188 | 0.3224 |
| 49 | 16.69 | 361 | Sucrose | 0.546 | 18.710 | 0.2991 |

**Table S4.** Plasma metabolites discriminating DME and non DME subjects based on metabolite profiling analyzed by GC-TOF-MS in extended cohort.

| **No.** | **RT**  **(min)** | **Unique**  **Mass** | **Metabolites** | **AUC** | **Fold Change (DME/non-DME)** | **t-test (*p*-value)** |
| --- | --- | --- | --- | --- | --- | --- |
| 110 | 10.23 | 246 | Glutamic acid | 0.733 | 0.671 | 0.0016 |
| 111 | 14.76 | 218 | Cystine | 0.665 | 0.732 | 0.0031 |
| 112 | 5.56 | 116 | Alanine | 0.526 | 0.777 | 0.2919 |
| 113 | 9.75 | 220 | Cysteine | 0.566 | 0.790 | 0.1990 |
| 114 | 9.45 | 232 | Aspartic acid | 0.676 | 0.814 | 0.0128 |
| 115 | 10.66 | 116 | Asparagine | 0.666 | 0.821 | 0.0113 |
| 116 | 11.12 | 156 | Glutamine | 0.643 | 0.834 | 0.0163 |
| 117 | 12.45 | 174 | Lysine | 0.686 | 0.864 | 0.0025 |
| 118 | 9.45 | 176 | Methionine | 0.588 | 0.883 | 0.0503 |
| 119 | 8.31 | 219 | Threonine | 0.600 | 0.887 | 0.1260 |
| 120 | 8.06 | 204 | Serine | 0.624 | 0.898 | 0.1099 |
| 121 | 12.58 | 218 | Tyrosine | 0.584 | 0.910 | 0.1026 |
| 122 | 11.72 | 142 | Ornithine | 0.587 | 0.913 | 0.2506 |
| 123 | 5.41 | 72 | Valine | 0.570 | 0.928 | 0.3203 |
| 124 | 10.33 | 218 | Phenylalanine | 0.592 | 0.940 | 0.2021 |
| 125 | 7.45 | 158 | Isoleucine | 0.556 | 1.047 | 0.3970 |
| 126 | 7.50 | 142 | Proline | 0.536 | 1.092 | 0.2950 |
| 127 | 12.64 | 333 | Galacturonic acid | 0.556 | 0.734 | 0.3916 |
| 128 | 13.64 | 441 | Uric acid | 0.738 | 0.757 | 0.0000 |
| 129 | 9.18 | 233 | Malic acid | 0.641 | 0.805 | 0.0152 |
| 130 | 11.76 | 273 | Citric acid | 0.684 | 0.835 | 0.0038 |
| 131 | 9.04 | 218 | Aminomalonic acid | 0.600 | 0.839 | 0.0915 |
| 132 | 5.05 | 174 | Pyruvic acid | 0.573 | 0.885 | 0.2182 |
| 133 | 7.80 | 189 | Glyceric acid | 0.615 | 0.906 | 0.4179 |
| 134 | 5.29 | 177 | Hydroxyacetic acid | 0.580 | 0.929 | 0.2498 |
| 135 | 7.32 | 299 | Phosphoric acid | 0.524 | 1.059 | 0.3006 |
| 136 | 5.70 | 133 | Hydroxylamine | 0.571 | 1.080 | 0.1967 |
| 137 | 7.06 | 189 | Urea | 0.621 | 1.210 | 0.0795 |
| 138 | 7.51 | 164 | Benzeneacetic acid | 0.719 | 1.291 | 0.0002 |
| 139 | 9.79 | 115 | Creatinine | 0.652 | 1.362 | 0.0382 |
| 140 | 6.12 | 191 | 3-Hydroxyisobutyric acid | 0.559 | 0.797 | 0.4210 |
| 141 | 13.03 | 311 | Palmitoleic acid | 0.620 | 0.806 | 0.2058 |
| 142 | 16.12 | 91 | Docosahexaenoic acid | 0.607 | 0.807 | 0.0408 |
| 143 | 14.20 | 339 | Oleic acid | 0.569 | 0.887 | 0.2840 |
| 144 | 14.18 | 337 | Linoleic acid | 0.551 | 0.905 | 0.3675 |
| 145 | 13.15 | 313 | Palmitic acid | 0.580 | 0.924 | 0.2076 |
| 146 | 15.35 | 131 | Oleamide | 0.543 | 1.042 | 0.3592 |
| 147 | 16.23 | 371 | Monopalmitin | 0.529 | 1.057 | 0.4039 |
| 148 | 8.86 | 229 | Decanoic acid | 0.615 | 1.124 | 0.2198 |
| 149 | 8.01 | 215 | Nonanoic acid | 0.639 | 1.140 | 0.0306 |
| 150 | 13.62 | 217 | myo-Inositol | 0.536 | 0.849 | 0.2777 |
| 151 | 7.25 | 117 | Glycerol | 0.562 | 0.930 | 0.2245 |
| 152 | 17.24 | 361 | Maltose | 0.593 | 1.160 | 0.3027 |
| 153 | 16.69 | 361 | Sucrose | 0.558 | 12.596 | 0.3025 |

**Table S5.** Plasma oxylipins identified in DME and non-DME subjects based on lipid profiling analyzed by HPLC-triple-Q-MS in extended cohort.

| **No.** | **RT**  **(min)** | **Molecular**  **weight** | **Product ion (*m/z*)** | **Metabolites** | **AUC** | **Fold Change (DME/non-DME)** | **t-test (*p*-value)** |
| --- | --- | --- | --- | --- | --- | --- | --- |
| 154 | 7.11 | 320.5 | 275.3 | 20-HETE | 0.639 | 0.090 | 0.0400 |
| 155 | 3.91 | 350.5 | 331.2 | 15-keto Prostaglandin E2 | 0.632 | 0.597 | 0.2465 |
| 156 | 7.29 | 320.5 | 301.25 | (±)5-HETE or (±)8-HETE | 0.654 | 0.645 | 0.0537 |
| 157 | 7.07 | 320.5 | 179.2 | (±)12-HETE | 0.631 | 0.674 | 0.0576 |
| 158 | 3.96 | 352.5 | 315.25 | 15-keto Prostaglandin F2α | 0.584 | 0.727 | 0.4015 |
| 159 | 8.02 | 318.5 | 203.25 | 5-OxoETE | 0.517 | 0.793 | 0.6130 |
| 160 | 7.14 | 320.5 | 163.3 | (±)5(6)-EET | 0.539 | 0.793 | 0.3446 |
| 161 | 3.72 | 354.5 | 309.25 | Prostaglandin F2α | 0.500 | 0.794 | 0.7950 |
| 162 | 4.08 | 496.7 | 177.1 | Leukotriene D4 | 0.510 | 0.804 | 0.4953 |
| 163 | 7.24 | 320.5 | 155.15 | (±)8(9)-EET | 0.527 | 0.851 | 0.5864 |
| 164 | 4.32 | 352.5 | 207.2 | 13,14-dihydro-15-keto Prostaglandin D2 | 0.570 | 0.857 | 0.7024 |
| 165 | 4.01 | 352.5 | 333.25 | 20-hydroxy Leukotriene B4 | 0.526 | 0.899 | 0.7728 |
| 166 | 7.00 | 320.5 | 167.2 | (±)11-HETE | 0.549 | 0.936 | 0.7367 |
| 167 | 4.94 | 336.5 | 317.3 | (±)12-HpETE | 0.501 | 0.939 | 0.7638 |
| 168 | 6.81 | 320.5 | 219.2 | (±)15-HETE | 0.509 | 0.971 | 0.8855 |
| 169 | 3.88 | 352.5 | 189.25 | Prostaglandin D2 | 0.552 | 0.971 | 0.8897 |
| 170 | 5.11 | 336.5 | 59.1 | 5(S)-HpETE | 0.587 | 0.981 | 0.9330 |
| 171 | 3.74 | 625.8 | 189.15 | Leukotriene C4 | 0.532 | 0.984 | 0.9494 |
| 172 | 7.28 | 320.5 | 257.25 | (±)11(12)-EET or (±)14(15)-EET | 0.553 | 1.014 | 0.9428 |
| 173 | 3.35 | 370.5 | 195.2 | Thromboxane B2 | 0.512 | 1.019 | 0.9622 |
| 174 | 3.25 | 370.5 | 163.2 | 6-keto Prostaglandin F1α | 0.521 | 1.023 | 0.9751 |
| 175 | 3.64 | 332.5 | 315.15 | Leukotriene A4 methyl ester | 0.530 | 1.041 | 0.7361 |
| 176 | 7.28 | 320.5 | 151.2 | (±)9-HETE | 0.524 | 1.189 | 0.6144 |
| 177 | 7.24 | 318.5 | 113.1 | 15-OxoETE | 0.715 | 1.282 | 0.0078 |
| 178 | 4.50 | 334.5 | 235.15 | Prostaglandin B2 | 0.565 | 1.312 | 0.5408 |
| 179 | 4.94 | 338.5 | 319.3 | (±)11(12)-DiHET or (±)14(15)-DiHET | 0.564 | 1.329 | 0.2971 |
| 180 | 6.07 | 318.5 | 299.85 | 12-OxoETE | 0.784 | 1.340 | 0.0015 |
| 181 | 4.48 | 334.5 | 315.25 | Prostaglandin A2 | 0.542 | 1.361 | 0.5007 |
| 182 | 5.17 | 336.5 | 195.2 | Leukotriene B4 | 0.564 | 1.399 | 0.2864 |
| 183 | 4.61 | 334.5 | 271.3 | Prostaglandin J2 | 0.590 | 1.414 | 0.2590 |
| 184 | 3.38 | 368.5 | 349.25 | 11-dehydro Thromboxane B2 | 0.554 | 1.563 | 0.3485 |
| 185 | 3.99 | 352.5 | 271.3 | Prostaglandin E2 or Prostaglandin H2 | 0.656 | 1.792 | 0.1153 |
| 186 | 4.50 | 354.5 | 113.1 | 13,14-dihydro-15-keto Prostaglandin F2α | 0.574 | 2.303 | 0.4475 |
| 187 | 3.98 | 352.5 | 221.25 | Lipoxin B4 | 0.554 | 2.311 | 0.2870 |
| 188 | 3.02 | 366.5 | 347.25 | 20-carboxy Leukotriene B4 | 0.755 | 5.198 | 0.0025 |
| 189 | 4.88 | 336.5 | 273.25 | 15(S)-HpETE | 0.571 | 15038095238.952 | 0.0734 |
| 190 | 7.39 | 344.5 | 141.15 | (±)7-HDHA | 0.549 | 0.786 | 0.3890 |
| 191 | 7.16 | 344.5 | 325.25 | (±)20-HDHA | 0.609 | 0.819 | 0.1458 |
| 192 | 7.38 | 344.5 | 189.25 | (±)8-HDHA | 0.618 | 0.823 | 0.2815 |
| 193 | 7.65 | 344.5 | 101.05 | (±)4-HDHA | 0.637 | 0.832 | 0.1426 |
| 194 | 7.06 | 344.5 | 233.25 | (±)16-HDHA | 0.577 | 0.878 | 0.4048 |
| 195 | 7.06 | 344.5 | 245.25 | (±)17-HDHA | 0.524 | 0.936 | 0.7492 |
| 196 | 7.24 | 344.5 | 153.15 | (±)10-HDHA | 0.514 | 1.012 | 0.9438 |
| 197 | 7.22 | 344.5 | 281.25 | (±)13-HDHA | 0.537 | 1.022 | 0.9048 |
| 198 | 5.01 | 360.5 | 153.2 | 10(S),17(S)-DiHDHA | 0.526 | 1.249 | 0.5587 |
| 199 | 6.54 | 318.5 | 179.2 | (±)12-HEPE | 0.608 | 0.736 | 0.1061 |
| 200 | 6.40 | 318.5 | 219.2 | (±)15-HEPE | 0.642 | 0.768 | 0.1897 |
| 201 | 6.52 | 318.5 | 299.25 | (±)18-HEPE | 0.587 | 0.893 | 0.4802 |
| 202 | 6.60 | 318.5 | 255.3 | (±)11(12)-EpETE | 0.591 | 0.906 | 0.5734 |
| 203 | 3.88 | 350.5 | 233.2 | Prostaglandin D3 | 0.541 | 1.609 | 0.4323 |
| 204 | 3.77 | 350.5 | 269.25 | Prostaglandin E3 | 0.548 | 1.709 | 0.3119 |
| 205 | 7.53 | 294.4 | 185.2 | 9-OxoODE | 0.711 | 0.677 | 0.0207 |
| 206 | 7.26 | 294.4 | 113.15 | 13-OxoODE | 0.607 | 0.583 | 0.0820 |
| 207 | 5.18 | 314.5 | 183.25 | (±)12(13)-DiHOME | 0.600 | 0.868 | 0.4094 |
| 208 | 5.30 | 314.5 | 201.2 | (±)9(10)-DiHOME | 0.600 | 0.872 | 0.4888 |
| 209 | 6.88 | 296.5 | 277.4 | (±)9(10)-EpOME | 0.558 | 0.908 | 0.5381 |
| 210 | 7.76 | 296.5 | 301.05 | (±)9-HODE or (±)13-HODE | 0.511 | 1.011 | 0.8072 |
| 211 | 6.15 | 294.4 | 275.25 | 9(S)-HOTrE | 0.587 | 1.023 | 0.9037 |
| 212 | 6.56 | 312.4 | 220.1 | 9(S)-HpODE | 0.526 | 1.090 | 0.6766 |
| 213 | 6.60 | 312.4 | 183.1 | 13(S)-HpODE | 0.623 | 1.100 | 0.4891 |

**Supplementary Figures**

**Figure S1.** Heat maps representing relative levels of metabolites between diabetic macular edema (DME) and non-DME subjects in discovery cohorts (A) and extended cohorts (B). *Left*―metabolites were selected based on VIP value (> 0.7) derived from PLS-DA analyzed by GC-TOF-MS. *Right*―metabolites derived from lipid profiling analyzed by HPLC-triple-Q-MS analysis. Asterisks indicate statistically significant metabolite differences between DME and non-DME groups (*p* < 0.05).


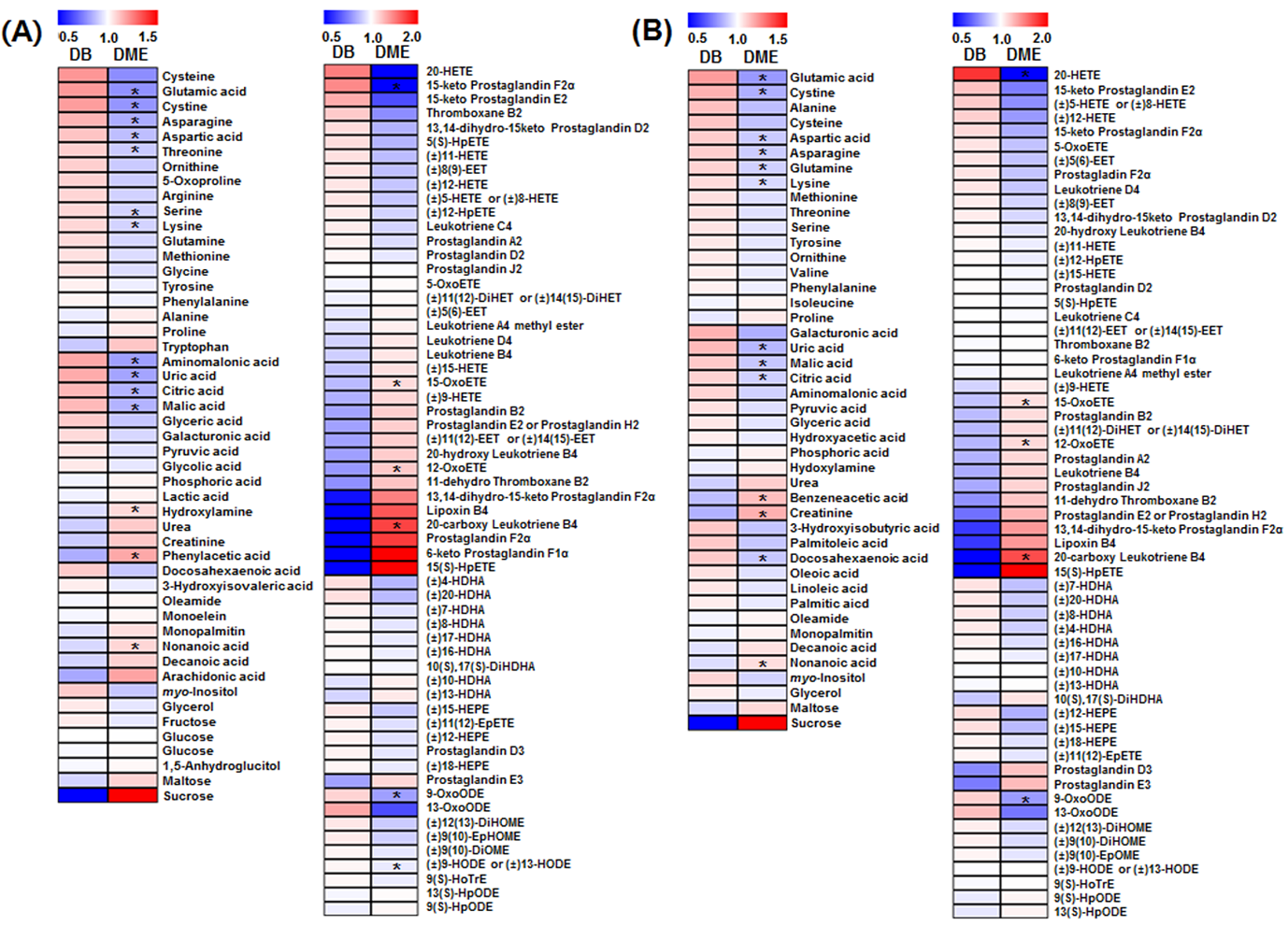


**Figure S2.** Principal component analysis (PCA) (A) and orthogonal partial least squares discriminant analysis (OPLS-DA) (B) score plots for plasma of DME and non-DME subjects analyzed by GC-TOF-MS in discovery cohort. ●―DME group, ●―non-DME group


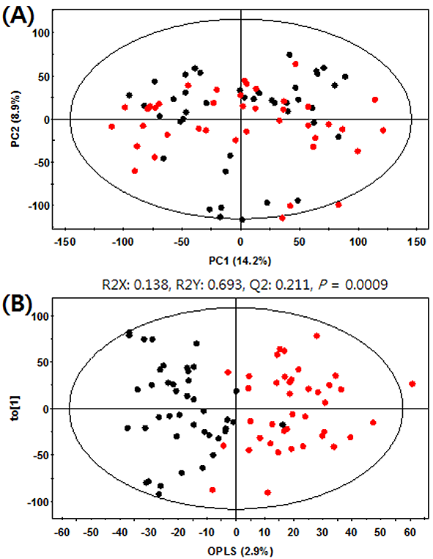


**Figure S3.** Combined receiver operating characteristic (ROC) curve of potential metabolite biomarkers distinguishing diabetic macular edema (DME) versus non-DME subjects in extended cohort. (A) identical 7 metabolites as selected in discovery cohort after GC-TOF-MS analysis-based metabolite profiling. (B) identical 4 oxylipins as selected in discovery cohort after lipid profiling analyzed by HPLC-triple-Q-MS. The combined ROC curves were overlain on single plots. The AUC values are shown inside the ROC curve.


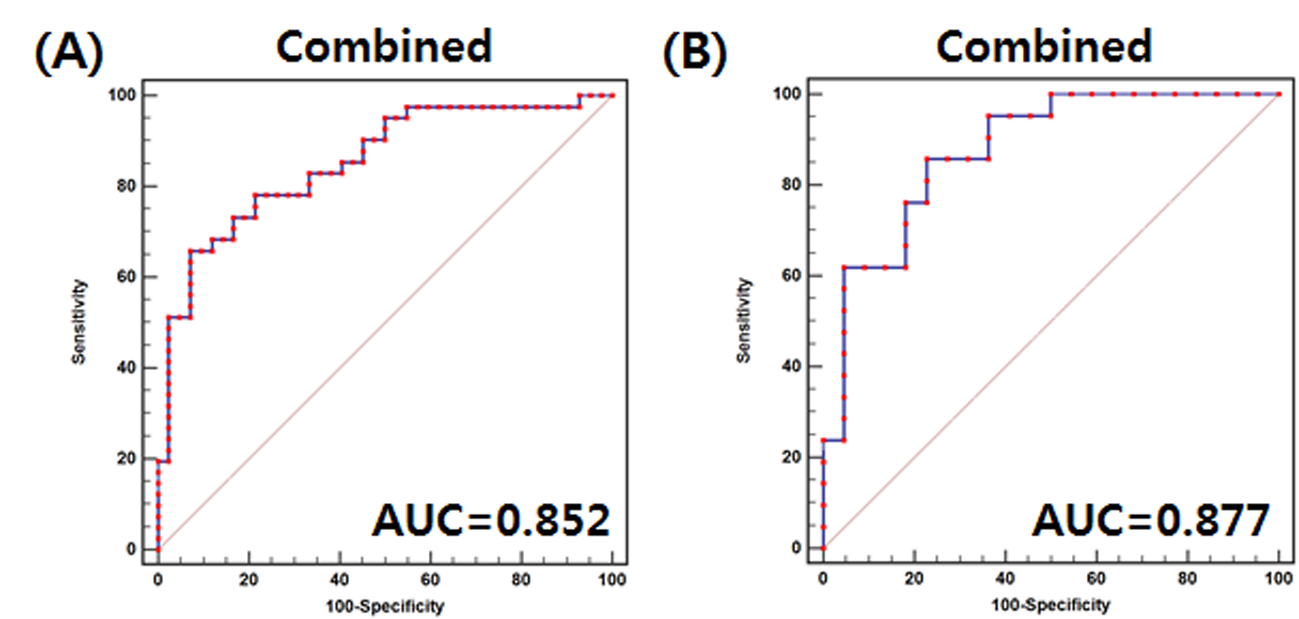

Supplement: Supplementary file 1 — Supplementary Information. [file 41598_2021_88104_MOESM1_ESM.docx]
